# Frequent Abdominal Pain in Childhood and Youth: A Systematic Review of Psychophysiological Characteristics

**DOI:** 10.1155/2014/524383

**Published:** 2014-03-13

**Authors:** Marco Daniel Gulewitsch, Judith Müller, Paul Enck, Katja Weimer, Juliane Schwille-Kiuntke, Angelika Anita Schlarb

**Affiliations:** ^1^Department of Psychology, Clinical Psychology and Psychotherapy, University of Tübingen, Schleichstraße 4, 72076 Tübingen, Germany; ^2^Department of Internal Medicine VI/Psychosomatic Medicine and Psychotherapy, University Hospital Tübingen, Frondsbergstraße 23, 72070 Tübingen, Germany; ^3^Faculty of Language and Literature, Humanities, Arts and Education (FLSHASE), University of Luxembourg, Route de Diekirch-B.P. 2, L-7201 Walferdange, Luxembourg

## Abstract

*Background*. Frequent abdominal pain (AP) in children and adolescents is often designated as functional gastrointestinal disorder. In contrast to research on psychological and social influences on the experience of AP in this population, psychophysiological features such as function of the autonomic nervous system, the central nervous system, or the endocrine system have rarely been studied. * Methods*. We conducted a systematic literature search for peer-reviewed journal articles referring to children with AP between 4 and 18 years. Studies on experimental baseline characteristics or reactivity of psychophysiological outcome parameters (autonomous nervous system, central nervous system, and endocrine parameters) were included. * Key Results*. Twelve of 18 included studies found psychophysiological differences between children with AP and healthy ones. These studies indicate a possible autonomic dysregulation and hypersensitivity of the central nervous system in children with AP following stimulation with stress or other intense stimuli. Mainly conflicting results were found regarding baseline comparisons of autonomic and endocrine parameters. * Conclusions and Inferences*. Frequent AP in children may be associated with an altered psychophysiological reaction on intense stimuli. It has to be considered that the current literature on psychophysiological characteristics of childhood AP is small and heterogeneous. In particular, multiparameter studies using validated experimental paradigms are lacking.

## 1. Introduction

Beside headache, frequently occurring abdominal pain (AP) is the most prevalent pain issue in children and adolescents [1]. It is often designated as idiopathic or as functional gastrointestinal disorder (FGID) [2] because a considerable proportion of children are lacking explanatory organic diseases [3, 4]. Current Rome III criteria on classification of FGIDs [2] comprise five AP related functional disorders: functional dyspepsia, irritable bowel syndrome (IBS), abdominal migraine, functional AP, and functional AP syndrome. Before the introduction of Rome criteria, the inconclusive term “recurrent abdominal pain” (RAP) was widely used. Apley and Naish [3] defined RAP as the presence of at least three episodes of AP over a period of 3 months, interfering with daily activities of the child. Although Rome classification is generally accepted by now, the use of diagnostic criteria has been inconsistent in the past. A large proportion of studies regarded children meeting Apley and Naish's criteria as a homogeneous group, but this limited its use for etiological research since there is evidence for distinct subgroups with specific symptom patterns [5]. In reviewing the subsequent articles we use the term “abdominal pain” (AP) instead and name applied criteria.

It is widely recognized that frequent AP is associated with increased psychological distress, especially with anxiety symptoms, anxiety disorders, or depression [6, 7]. Beyond psychological distress, frequently occurring AP is related to increased functional impairment in everyday life [8], school absence [9], and health-care utilization [9]. Dealing with daily hassles might be a crucial psychological factor: children with AP report more daily stressors than healthy children and daily stressors predicted somatic symptoms in children with AP stronger than in healthy ones [10]. Experiencing AP is reported to be linked to different patterns of stress appraisal and coping behavior [11].

Chronic stress is associated with psychophysiological alterations in the autonomic nervous system and the endocrine system [12], but, in contrast to research on psychological and social correlates in children with AP, psychophysiological features have rarely been studied in this population. Psychophysiology is aimed at physiological responses which are supposed to be associated with states such as anxiety, depression, stress, or pain. Prominent domains are the function of the autonomic nervous system, the endocrine system, and the central nervous system. The investigation of these features in children with AP could contribute to the understanding of the complex relationship of psychological and physiological influences on the development and maintenance of this disorder. With this review, we aim to outline the current state of research regarding psychophysiological features of frequent AP in children and adolescents.

## 2. Methods

### 2.1. Paper Search and Evaluation

For the purpose of this review, we searched PUBMED, PsychINFO, and Web of Science for published peer-reviewed journal articles in English or German with variable combinations of keywords (see Table 1). In accordance with Rome III criteria we comprised studies of children and adolescents aged 4–18 years because neonates and toddlers feature divergent functional gastrointestinal symptoms [13].

Inclusion criteria were (1) at least one identifiable group of children or adolescents with frequent AP aged 4–18 years; (2) at least one psychophysiological parameter (see Table 1) as outcome variable/dependent variable measuring baseline activity or reactivity upon stimuli; (3) experimental study design and report of data based on means and in statistical comparison to a control group.

**Figure 1:**
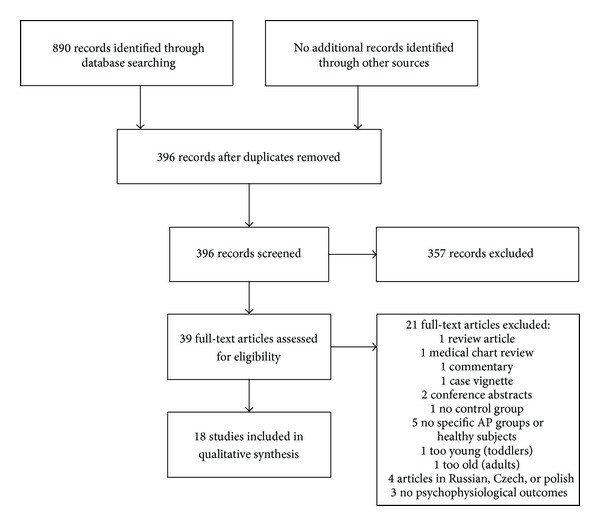
Procedure and results of the paper search and evaluation.

Procedure and results of the paper search and evaluation are depicted in Figure 1. Our search strategy yielded three domains. Subsequently, we refer to studies on the autonomic nervous system (ANS), the central nervous system (CNS), and the endocrine system (ES). The literature search was completed in January 2013.

## 3. Results

As shown in Figure 1, a total of 890 records were identified through database searching whereby 396 records remained after duplicates were removed. Manual screening of the titles and the abstracts resulted in the exclusion of 357 records. The remaining 39 full-text articles were assessed for eligibility whereof 21 were excluded since they did not fulfill inclusion criteria for this review (reasons are listed in Figure 1). Eighteen studies were finally included in qualitative synthesis. Twelve studies included parameters of the ANS, four included parameters of the CNS, and four included parameters of the ES. Three studies referred to more than one domain of psychophysiological parameters. An overview of studies is listed in Table 2.

### 3.1. ANS Function: Pupillary Reflex

The first study on ANS function in children with AP was carried out by Rubin and colleagues in 1967 [14] and addressed the pupillary reflex which is controlled by parasympathetic (constriction) and sympathetic (dilatation) influences. The authors employed pupillary reactivity in 13 AP children (daily to weekly pain frequency for at least half a year; mean age 10.6 years) and 12 asymptomatic controls. The pupil size was measured during rest, during a cold pressor task, and during a 15 min recovery period. No significant difference between the groups could be found at baseline and during cold pressor task. After the cold pressor task, children with AP showed a slower decrement in pupil size and required 6.5 min to attain the former pupil size while this was achieved in healthy children after one minute. This was the first study to show an aberrant ANS recovery after stress.

Another early study by Apley and colleagues [15] also compared pupillary reactions in 16 children with AP (mean age 9.8 years), 14 children with emotional problems, and 20 controls at rest and in response to a cold pressor task. At rest and during the cold pressor task, children with AP did not differ from the other groups. After stress, an unstable pupillary recovery pattern (repetitive dilation and constriction) was found in children with AP and in children with emotional problems. The pupil sizes of the AP group did not return to the resting level while the pupil sizes of the other groups did and even became smaller.

Battistella and colleagues [16] also applied pupillography to compare the pupil diameter of 18 children with AP (mean age 12.8 years) and 15 age-matched healthy controls. AP was defined according to Apley and Naish's criteria with the presence of additional autonomic symptoms during pain (e.g., vomiting, nausea, or pallor). Pupillary diameter was assessed before and several times after applying phenylephrine eye-drops to the right eye. Before instillation, the pupillary diameter was almost identical in both groups. After the stimulation, dilatation was greater in children with AP. Although this comparison did not reach significance, the authors concluded a potentially disturbed receptor sensitivity in the iris neuromuscular junction which may be caused by a sympathetic hypofunction. The three early studies on pupillography are not conform to today's standards but generated first hypotheses on autonomic dysfunction.

### 3.2. ANS Function: Heart Rate, Blood Pressure, and Skin Conductance

The 1982 study by Feuerstein et al. [17] was the first to include measurements of heart rate (HR), vasomotor reaction (digital blood volume pulse), and an additional forearm electromyogram (EMG) in children with AP. Ten children with AP according to Apley and Naish's criteria (9–14 years), 9 children with chronic non-pain-related conditions, and 10 healthy children were compared regarding their reaction to a cold pressor task. Feuerstein and colleagues could not show any significant differential responses between the groups with regard to HR, vasomotor reaction, or the EMG. The authors discussed whether the cold pressor task might have been a too strong stimulus and prone to ceiling effects. Their choice of parameters prevented results regarding the parasympathetic response of the ANS.

A study by Dufton and colleagues [18] aimed to investigate the HR reactivity in a laboratory setting. They included 21 children with AP according to Apley and Naish's criteria (mean age 11.1 years) from a tertiary care gastroenterology clinic, 21 children with anxiety disorders, and 21 healthy control children. One-third of children with anxiety disorder met Apley and Naish's criteria. Therefore children with AP and those with anxiety were combined to one “clinical” group. Groups were compared at five time points (baseline, serial subtraction task, social stress interview, cold pressor task, and recovery). The “clinical” group displayed significantly higher HRs during baseline and cold pressor task indicating a greater sympathetic arousal. Whereas the reactivity paradigm is a strength of the study, the heterogeneity of the “clinical” group and the use of only one psychophysiological parameter (HR) limit the validity of the study.

A pilot study regarding multiparameter stress reactivity by Dorn and colleagues [19] conducted the Trier Social Stress Test for Children with 14 AP children (Apley and Naish's criteria; mean age 12.7 years) in comparison to 14 children with anxiety disorders and 14 healthy controls. The baseline HR did not differ significantly between the three groups and no group differences in maximum HR were observed during the tasks. The AP group showed a trend for higher levels of baseline systolic blood pressure than the control group. Children with AP and with anxiety had similar levels. The multiparameter approach and the standardized social stress induction are strengths of the study. Unfortunately, the authors did not perform their calculations based on repeated measures. The design lacks consistent assignment criteria of participants to both symptomatic groups which led to a substantial overlap of categories. Furthermore, the small sample size was a limitation.

A study by Bakker and colleagues [20] investigated primarily the auditory startle reflex (CNS) in children with AP which is discussed further below. Additionally, the sympathetic skin response was recorded during presentation of auditory stimuli. Twenty children (mean age 12.4 years), classified as functional AP (*n* = 13) or irritable bowel syndrome (IBS) (*n* = 7) according to Rome III, and 23 healthy children participated. The authors observed a trend for a larger sympathetic skin response during the experimental task in children with AP. Up to now, this is the only study on reactivity of the sympathetic skin response upon stimuli.

### 3.3. ANS Function: Heart Rate Variability

Analysis of heart rate variability is used to gain information about the activation of the sympathetic and the parasympathetic branch of the ANS. In doing so, frequency-based power spectral analysis of the interbeat intervals allows extracting the oscillatory components of the RR intervals. The high-frequency component (HF) which occurs at 0.15–0.4 Hz is associated with the vagal regulation of respiratory sinus arrhythmia and indicates parasympathetic activity. Low-frequency oscillation (LF) between 0.04 and 0.15 Hz is thought to reflect sympathetic activation although the influence of both sympathetic and parasympathetic activity on the LF band is discussed [21]. The LF/HF ratio is often referred to as an index of sympathovagal balance.

The first study reporting heart rate variability (HRV) measurement in children with AP was conducted by Olafsdottir and colleagues [22] in 2001. Twenty-five children (mean age 10.7 years) with AP according to the criteria by Apley and Naish were compared to 23 healthy controls regarding respiratory sinus arrhythmia (RSA) and sympathetic skin conductance. RSA is considered as an index of parasympathetic ANS activity and was assessed during a deep breathing task with explicit respiration frequency. The study included only baseline measurement and has to be regarded as a pilot study. No significant baseline differences were found in both RSA or skin conductance. The authors discussed that the participants' complaints may not have been sufficiently chronic to produce a persistent autonomic dysfunction at rest which may be observed under the influence of stress.

HRV assessment using 24 h monitoring is a robust and naturalistic method for assessing general ANS function at day and night. Sowder and colleagues [23] compared the autonomic regulation between 20 children with AP (not specified classification “functional AP”; mean age 12.6 years) and 10 healthy controls by the means of long-term HRV. HRV, operationalized as sympathovagal balance, was analyzed frequency-based as low-to-high frequency ratio (LF/HF). Additionally, the HR time based measures NN50 and pNN50 were calculated which gather changes in consecutive normal sinus intervals exceeding 50 ms and reflect mostly the influence of parasympathetic activity. The study describes a HRV biofeedback training but we refer only to baseline comparisons. A significantly lower (parasympathetic) HF activation and a higher baseline LF/HF ratio were reported for children with AP indicating an autonomic dysregulation in terms of a vagal withdrawal (parasympathetic deactivation). This was supported by significant group differences regarding the time-based HRV measures. The control group was small and only little information was provided on the sample characteristics and the recruitment setting which narrows the validity of the results.

The most recent study by Jarrett and colleagues [24] also used long-term HRV monitoring to compare 100 7–10-year-old children with functional AP or IBS (according to Rome III) to 62 healthy controls. Children with AP were recruited in primary and tertiary care settings. HRV analysis, based on 24 h ECG, included the frequency-based HF component and LF/HF ratio. In this well-powered and methodologically sound study, the HRV measures did not differ significantly between the groups and therefore did not imply a general ANS dysregulation in children with AP. The authors emphasized explicitly that the results should not be extrapolated to an adolescent population. Exploratory analyses suggested a negative correlation between parasympathetic activity and psychological distress in girls with AP.

Puzanovova and colleagues [25] conducted one of the few studies investigating the reactivity of HRV parameters. Recruited from tertiary care, they compared 45 children with AP according to Apley and Naish's criteria (mean age 11.6 years) with 22 healthy controls regarding HRV during a 5-minute resting baseline and manipulated success/failure in a cognitive task. During baseline the two groups did not differ with respect to HRV. In the success condition, children with AP showed significantly elevated levels of LF and HF (coactivation) whereas healthy controls exhibited no change. In the failure condition, no significant differences between the two groups could be found. The authors concluded that successful task performance seemed to have produced a coactivation of sympathetic (LF) and parasympathetic (HF) activity in children with AP which may suggest an increased vigilance and anticipation of potential failure. The nonsignificant differences between resting period and failure may be due to the fact that the task was not sufficiently stressful to activate a response. It cannot be ruled out that psychological characteristics such as trait anxiety are responsible for the observed coactivation of sympathetic (LF) and parasympathetic (HF) activity.

Friesen and colleagues [26] investigated the HRV reactivity of 9 children suffering from Rome III functional dyspepsia (FD; mean age 13 years) following a test meal or rapid water loading. They were compared to 28 healthy controls. Sympathovagal balance (LF/HF) was assessed. Additionally, an instability coefficient (IC) was calculated to detect short-lasting frequency variations whereas the clinical significance of this parameter remained unclear. Before the meal, the groups did not differ regarding resting LF, HF, and LF/HF but did so regarding the IC. No significant change in HRV parameters from before to after the meal was found in children with FD whereas controls showed decreasing HF and increasing LF and LF/HF. After the meal, HF was significantly elevated in FD children compared to healthy controls whereas LF, LF/HF, and IC were significantly lowered. The rapid water loading test (on another day) revealed no HRV differences between the groups. One may conclude that FD is associated with a loss of shift in autonomic balance which is found in healthy children after a solid meal. The study is underpowered and a transfer to other AP-related FGIDs is pending. Symptom provocation by ingestion may be a promising approach for future studies.

### 3.4. CNS Function: General Abnormalities

Apley and colleagues were the first who conducted EEG measurement in children with AP [27]. A large sample of 133 children with AP according to Apley and Naish's criteria and 133 healthy children (age range of the complete sample 3–14 years) was examined in terms of epileptiform abnormalities (“masked epilepsy”). The EEG recording of resting and evoked responses (hyperpnoea and photic stimulation) did not show an association between AP and EEG abnormalities.

### 3.5. CNS Function: Hypersensitivity to Sensory Stimuli

A study by Bakker and colleagues [20] examined whether children with AP have a generalized hypersensitivity for nonvisceral stimuli using an auditory startle paradigm. Twenty children (mean age 12.4 years) classified as functional AP or IBS according to Rome III and 23 healthy children participated. Additionally, 25 children with anxiety disorders but without AP were included. The multiple muscle auditory startle reflex, measured over six left-sided muscles by response probability and by EMG magnitude (blink response), was significantly enlarged in patients with AP compared to control subjects but did not differ from patients with anxiety disorder. Comorbid anxiety disorders among children with AP did not affect the auditory startle reflex. Based on results of the auditory startle paradigm, the authors found increased responses after nonvisceral sensory stimuli in children with AP-related FGID which points towards an abnormally sensitive CNS in general.

Hermann and colleagues [28] investigated 14 children with AP (Apley and Naish's criteria; mean age 12.1 years) and 15 controls in an attention task in which they had to response to rare target tones and ignore painful or nonpainful mechanical stimuli at the fingertip. An EEG was recorded and N1, P2, and P3 components of somatosensory-evoked potentials were compared between the groups. No significant group differences could be shown for the early components N1 and P2, but children with AP exhibited altered central processing in terms of a shorter latency and a larger P3 amplitude following painful and nonpainful stimuli. This may represent a general attentional bias of an automatic focus on somatic sensations which has a high explanatory value. A stronger focus on somatic sensations may underlie increased levels of somatic symptoms whereas the exact relationship between hypersensitivity and attentional bias to body sensations has to be elucidated in more detail.

Seino and colleagues [29] examined brainstem auditory evoked potentials (BAEP) following click sounds in 141 seven-year-old children. Seventy-five of them had at least one of seven GI symptoms during the last two weeks (AP was one of these symptoms) and were contrasted to children without GI symptoms during the two weeks. BAEP latencies are an indicator of sensory processing and can be used to estimate CNS responsiveness. The authors found shorter latencies of a specific component (wave III) in symptomatic girls but not in boys. This may be due to altered perceptual responses to afferent signals and points towards a hypersensitivity to environmental stimuli in girls (but not in boys which could not be explained). Since this study focused on several GI symptoms (not exclusively on AP) and did not apply established criteria, its validity for FGIDs in general is limited.

### 3.6. Endocrine Functions: Concentrations of Oxytocin, Cortisol, and Prolactin

Alfvén and colleagues [30] investigated the plasma concentrations of oxytocin, cortisol, and prolactin in 40 children with AP according to Apley and Naish's criteria (mean age 10 years). They were compared to 34 controls which were free of functional complaints but partially had other organic diseases. The blood sample was collected after an overnight fast period. Oxytocin and cortisol levels were significantly reduced in children with AP and both values persisted at a second examination three months later. Prolactin levels did not differ. Oxytocin influences vagal nerve activity and may provide a hormonal link to autonomic dysregulation. The heterogeneous control group, including allergic and inflammatory diseases, aggravates the interpretation.

A second study by Alfvén [31] studied plasma oxytocin after an overnight fast period. They compared 32 children with “psychosomatic” AP according to Apley and Naish's criteria (mean age 9.6 years) with 15 children suffering from AP because of inflammatory bowel disease (IBD) and 79 healthy controls. Children with AP showed significant lower plasma oxytocin concentrations than healthy controls but did not differ from IBD children. A subgroup of 23 children with AP and 21 healthy controls were additionally compared regarding plasma cortisol. The difference between the groups did not reach significance with AP children exhibiting lower cortisol concentrations. Low levels of cortisol and oxytocin may point towards an adaption of the organism to chronic stress [32]. It remains unclear whether this endocrine pattern is innate or acquired. The results have to be interpreted with caution because a single serum cortisol estimation might be liable to individual and diurnal variations. The group sizes and probably heterogeneous AP samples limit extensive interpretations.

The two previous studies were “snapshots” at one measurement point. As the concentration of cortisol changes considerably during the day, Törnhage and Alfvén [33] inspected the diurnal variation in 31 children with AP according to Apley and Naish's criteria (mean age 10.8 years) and 306 healthy children. Saliva samples were collected in the morning, at noon, and in the evening. The AP group presented significantly higher morning saliva cortisol values compared to the healthy group but not at noon or in the evening. Beyond that, children with AP showed an increased total secretion, calculated as area-under-the-curve, during the entire morning period (8:00–13:00 h). The authors stated that high cortisol concentrations might be associated with a higher proportion of children suffering from depression in this sample, but this remained speculative because respective parameters were not assessed. Another explanation the authors offer is that awakening might be an especially stressful event for children with AP. This could be reflected in free (saliva) cortisol but probably not in total serum concentration.

A multiparameter study on stress reactivity of children with AP by Dorn and colleagues [19], mentioned earlier in this text, conducted a laboratory social stress protocol with 14 children with AP (Apley and Naish's criteria) in comparison to 14 children with anxiety disorders and 14 healthy children. Six saliva cortisol samples were obtained throughout the protocol. Dorn and colleagues could not find differences between the groups regarding cortisol at baseline or maximum cortisol values. Beyond other methodological issues such as small sample size or inconsistent group assignment, calculations of area-under-the-curve values for total cortisol release were not conducted.

## 4. Discussion

A number of potential mechanisms may underlie frequent childhood AP. Research in the last decades focused primarily on psychological and social influences on the development and maintenance of childhood AP. A multicausal biopsychosocial perspective has been suggested and is widely applied by now, but this attempt lacks profound knowledge about the underlying biological components.

Previous studies confirmed that frequent AP is associated with anxiety [6], parental behavior [34], and stress coping [11]. However, it remained unclear which mechanisms link these factors to the extensive experience of pain. Our analysis of included studies revealed a variety of different methodological attempts to assess psychophysiological features and reactions of children with frequent AP. Most studies referred to ANS function (12 articles) while studies exploring the role of the endocrine system (4 articles) and the CNS (4 articles) were rare.

### 4.1. Function of the Autonomic Nervous System

Investigation of ANS estimates of children with frequent AP is reasonable since the ANS is fast reacting to stress and there are studies which point to autonomic abnormalities in adults with IBS [35]. Three early studies with AP children addressed ANS function by examining pupillary reactivity and they only allow vague conclusions about pathophysiological mechanisms. Studies attending cardiovascular function as an index of sympathetic arousal (HR, blood pressure) showed inconsistent results. Three studies also reported HR reactivity following stress-inducing tasks (cold pressor task [17, 36] or social stress [36]). Only the study by Dufton and colleagues [36] could show greater sympathetic arousal in terms of elevated HR during the cold pressor task. It has to be noted that all three studies lack in methodological issues, above all, the considerable overlap of AP and anxiety in their compared groups. Beyond that, HR change over time as only parameter is an imperfect measure for autonomic function. High HR may be associated with high stress or anxiety but can also point to autonomic flexibility if HR returns fast to baseline [37]. This makes clear that such studies should include multiple ANS parameters (e.g., skin conductance, blood pressure, and HRV-based parameters). Skin conductance as a measure for sympathetic activation was used in only two studies with ambiguous results [20, 22].

Most studies addressing the ANS used HRV assessment which is a generally accepted tool for estimating ANS function. Particular attention was paid to the activity of the vagal nerve which is mostly assessed by parasympathetic HRV components (HF or RSA) and has influence on short-term HR adjustments. Hence it is often designated as an index for autonomic flexibility. The LF/HF ratio is mostly considered as an estimate of sympathovagal balance. A shift in sympathetic/parasympathetic balance towards sympathetic dominance may be caused by vagal withdrawal or by sympathetic dominance. But interpretation of the LF component and therefore the LF/HF ratio as measure of the sympathetic branch must be done with caution [21].

While paced deep breathing to measure RSA has only been used in one study including children with AP (yielding no group difference) [22], most studies applied frequency-based analyses. Only the study by Sowder and colleagues [23] could show baseline LF/HF differences in adolescents. Alterations of baseline HRV parameters were also found in adults with IBS using long-term HRV recordings covering 24 h. For example, Heitkemper and colleagues [38] used 24 h HRV recording and found that adult IBS patients featured a shift in sympathetic/parasympathetic balance (LF/HF ratio) towards sympathetic dominance which was caused by decreased HF power (vagal withdrawal). Burr and colleagues also reported alterations of the vagal tone (HF band) in women with IBS [39]. They found that the vagal component of a 24 h HRV measurement appeared to be reduced in women with severe AP. These results refer to a baseline autonomic dysfunction in at least part of adults with IBS.

Regarding children with AP, Puzanonva and colleagues [25] and Friesen and colleagues [26] found differentiating HRV reactivity patterns following cognitive challenge or meal ingestion. In adults with IBS, only one study investigated the reactivity of the HRV. Elsenbruch and colleagues [40] applied a mental stressor (reaction time task with noise “punishment” based on Stroop color test) in 24 women with IBS and 20 controls. Women with IBS demonstrated no differential HRV response. It should be noted that the stress induction might have been too weak to achieve a differential effect since it did not include social component (personal relevance).

Current studies regarding ANS function in children with AP are difficult to compare because of different inclusion criteria, different paradigms, and heterogenic analysis procedures. Future studies should avoid wide age ranges since vagal activity is influenced by maturation [41]. Taken together, it becomes obvious that there are some indicators of altered function of the ANS whereas other studies did not find group differences. Current state of research in ANS function is lacking in multiparameter reactivity studies using validated experimental paradigms. Vagal nerve activity is known to regulate GI motility and vagal withdrawal is associated with decreased bowel contractions, reduced motility, constipation, and pain whereas high vagal activity can lead to increased contractions and diarrhea [42]. Abnormal gastrointestinal motility has been identified in only a few studies in children with AP [43, 44] and should be further investigated in combination with ANS measures.

### 4.2. Function of the Central Nervous System

Four studies addressed the CNS function of children with AP. Of these, one described baseline EEG measurement [27], one investigated the auditory startle reflex [20], one examined somatosensory-evoked potentials in reaction to stimuli [28], and one focused on brainstem auditory evoked potentials [29]. Despite the use of different paradigms and methods, the last three studies were based on the underlying idea of CNS hypersensitivity to sensory stimuli. These studies indicate a possible CNS hypersensitivity in at least parts of the samples. This may be an underlying factor of visceral and generalized extraintestinal hyperalgesia that has been found in children with AP [45, 46]. However, studies on visceral and extraintestinal hyperalgesia were not included in this review since they do not feature psychophysiological parameters as outcome variables, but reduced pain thresholds may play an important role in the maintenance of childhood AP. Beyond CNS hypersensitivity it has been hypothesized that adrenaline-stimulated vagal feedback is responsible for changes in visceral pain perception which might be a consequence of autonomic dysregulation [47]. Up to now, only one study with AP children could demonstrate lowered extraintestinal pain thresholds after stimulation of the ANS by social stress [36].

### 4.3. Endocrine Functions

Four studies investigated the endocrine system in children with AP. The findings are inconsistent but highlight altered concentrations of oxytocin and cortisol. These two hormones were reduced in blood plasma after an overnight fasting period; in addition, free cortisol was elevated in saliva during the morning-to-noon period. Reported studies followed several paradigms: baseline differences, awakening response, diurnal rhythm, or reactivity on stressors. Cortisol concentration is strongly related to chronic stress and it remains unclear whether the endocrine patterns found are innate or acquired. Not every referenced study used up-to-date analysis methods (e.g., area-under-the-curve) and, furthermore, there are limitations regarding sample structure and size. Additionally, there may be crucial differences in measuring free saliva cortisol and plasma cortisol [48]. In adults suffering from IBS, increased and blunted levels of cortisol have both also been found which might be explained by psychiatric comorbidities, different IBS subtypes (diarrhea- or constipation-predominant), or alterations of diurnal cortisol secretion [49, 50]. Future studies with AP children should analyze awakening response and diurnal rhythms as well as their reactivity to stressors. Beyond this, the influences of gender, chronicity, and mental disorders on endocrine function should be considered in more detail.

### 4.4. Conclusions and Perspective

Regarding the reviewed studies, it is uncertain whether the recruited samples of children with AP represent one homogenous group. Rome classification is the up-to-date approach to distinguish subgroups of children with frequent AP without organic origin [2]. However, most of the reported studies were not based on Rome classification and did not discriminate between different symptom patterns (e.g., changes of stools). This prompts questions regarding the validity of results. Severity of symptoms may be associated with different psychophysiological findings which could be demonstrated in adults with IBS [35]. Up to now, it is unclear if this also applies to children with frequent AP. Additionally there is a risk of confounding effects concerning psychiatric comorbidity. There is a strong need to separate AP children with and without comorbid disorders in future study designs. The setting of the study should be clearly stated and kept in mind when interpreting results. Studies using selected populations in hospitals and other secondary and tertiary care facilities may not be representative for community children with frequent AP.

Whereas the cross-section nature of studies partially suggests altered psychophysiological functioning, it is unclear whether this is cause or result of frequent AP or even an epiphenomenon. Studies examining course and reversibility of psychophysiological alterations are lacking. To answer these questions, multiparameter longitudinal studies with sufficient sample sizes will be needed.

## Figures and Tables

**Table 1:** Variable combination of English keywords used for paper search.

Children OR adolescents	AND *abdominal pain OR irritable bowel syndrome OR dyspepsia OR	AND psychophysiology OR oxytocin OR cortisol OR blood pressure OR endocrine* OR reactivity OR heart* OR *nervous system OR Hypothalamic-pituitary-adrenal axis/HPA OR electrodermal activity/EDA OR skin conductance OR evoked potentials OR electroencephalography, EEG OR startle

*is meant as symbol for a wildcard.

**Table 2:** Characteristics of included studies (ordered by publication date).

First author, Year	Psychophysiological parameters	Experimental paradigm	Inclusion criteria	*N* AP group	*N* controls	Age AP group (range) in years
Apley, 1956 [27]	CNS: EEG (not specified)	EEG recording of resting and evoked responses (hyperpnoea and photic stimulation)	Apley and Naish's criteria	133	133 healthy	NN (3–14*)

Rubin, 1967 [14]	ANS: Pupillary diameter	Reactivity on cold pressor task	More than 6 months, at least weekly frequency	13	12 healthy	10.6 (8–14)

Apley, 1971 [15]	ANS: Pupillary diameter	Reactivity on cold pressor task	Not specified (recurrent AP)	16	14 healty 20 emotional disturbance	9.8 (5–13)

Battistella, 1992 [16]	ANS: Pupillary diameter	Reactivity on phenylephrine eye-drops	Apley and Naish's criteria + dysautonomic symptoms	18	15 healthy	12.8 (10–16)

Feuerstein, 1982 [17]	ANS: HR, digital blood volume pulse EMG	Reactivity on cold pressor task	Apley and Naish's criteria	10	9 organic ill 10 healthy	NN (9–14)

Alfvén, 1994 [30]	ES: blood plasma oxytocin, cortisol, and prolactin	Blood plasma concentrations of hormones after an overnight fast period	Apley and Naish's criteria	40	34 organic ill	10 (7–16)

Olafsdottir, 2001 [22]	ANS: HRV (RSA via deep breathing test), EDA	Baseline comparison	Apley and Naish's criteria	25	23 healthy	10.7 (7–15)

Dorn, 2003 [19]	ANS: HR, SBP ES: salivary cortisol	Reactivity on mental and social stress (Trier Social Stress Test)	Apley and Naish's criteria	14	14 healthy 14 anxiety	12.7 (NN)

Alfvén, 2004 [31]	ES: blood plasma cortisol	Blood plasma concentrations of hormones after an overnight fast period	Apley and Naish's criteria	23	21 healthy	9.5 (NN)
	ES: blood plasma oxytocin	Blood plasma concentrations of hormones after an overnight fast period	Apley and Naish's criteria	32	15 IBD 79 healthy	9.6 (6–15*)

Törnhage, 2006 [33]	ES: salivary cortisol	Diurnal variation of salivary cortisol	Apley and Naish's criteria	31	306 healthy	10.8 (6–18)

Hermann, 2008 [28]	CNS: EEG N1, P2, and P3	Attentional bias to painful and innocuous somatic stimuli	Apley and Naish's criteria	14	15 healthy	12.1 (10–15)

Puzanovova, 2009 [25]	ANS: HRV (frequency-based)	Reactivity on success/failure in a cognitive task	Apley and Naish's criteria	45	22 healthy	11.6 (9–16*)

Bakker, 2010 [20]	CNS: EMG ANS: EDA	Auditory startle paradigm	Rome III FAP or IBS	20	23 healthy 25 anxiety	12.4 (NN)

Friesen, 2010 [26]	ANS: HRV (time- and frequency-based)	Reactivity following a test meal or rapid water loading, respectively	Rome III FD	9 resp. 8	28 healthy	13 (11–17)

Sowder, 2010 [23]	ANS: HRV (time- and frequency-based)	Baseline comparison	Not exactly specified (most probable Apley and Naish's criteria)	20	10 healthy	12.6 (5–17)

Dufton, 2011 [18]	ANS: HR	Reactivity on mental and social stress, cold pressure task	Apley and Naish's criteria	21	21 healthy 21 anxiety	11.1 (8–16*)

Jarrett, 2012 [24]	ANS: HRV (frequency-based)	24 h monitoring	Rome III FAP or IBS	100	62 healthy	8.9 (7–10)

Seino, 2012 [29]	CNS: EEG latencies of I wave, III wave, IV wave, and V wave	Brainstem auditory evoked potentials following click sounds	Presence of at least one of seven GI symptoms in the last two weeks	75	66 without GI symptoms	7 (all at the same age)

ANS: autonomic nervous system, AP: abdominal pain, CNS: central nervous system, EDA: electrodermal activity, EEG: electroencephalography, EMG: electromyography, ES: endocrine system, FAP: functional abdominal pain, FD: functional dyspepsia, GI: gastrointestinal, HR: heart rate, HRV: heart rate variability, IBD: inflammatory bowel disease, IBS: irritable bowel syndrome, NN: not named, SBP: systolic blood pressure.

*Refers to the complete sample or to the sample prior to group assignment.
